# Cardiovascular Disease Rates, Outcomes, and Quality of Care in Ontario Métis: A Population-Based Cohort Study

**DOI:** 10.1371/journal.pone.0121779

**Published:** 2015-03-20

**Authors:** Clare L. Atzema, Saba Khan, Hong Lu, Yvon E. Allard, Storm J. Russell, Mario R. Gravelle, Julie Klein-Geltink, Peter C. Austin

**Affiliations:** 1 Institute for Clinical Evaluative Sciences, Toronto, Ontario, Canada; 2 Sunnybrook Health Sciences Centre, Toronto, Ontario, Canada; 3 University of Toronto, Toronto, Ontario, Canada; 4 The Métis Nation of Ontario, Ottawa, Ontario, Canada; Chinese Academy of Medical Sciences, CHINA

## Abstract

**Background:**

The burden of cardiovascular disease in the Métis, Canada’s fastest growing Aboriginal group, is not well studied. We determined rates of five cardiovascular diseases and associated outcomes in Ontario Métis, compared to the general Ontario population.

**Methods:**

Métis persons were identified using the Métis Nation of Ontario Citizenship Registry. Métis citizens aged 20–105 were linked to Ontario health databases for the period of April 2006 to March 2011. Age- and sex-standardized prevalence and incidence of acute coronary syndromes (ACS), congestive heart failure (CHF), cerebrovascular disease (stroke), atrial fibrillation, and hypertension were compared between the Métis and the general population. Secondary outcome measures included one-year hospitalizations and mortality following the incident cardiovascular diagnosis, as well as quality-of-care measures.

**Results:**

There were 12,550 eligible Métis persons and 10,144,002 in the general population. The adjusted prevalence of each disease was higher (p<0.05) among the Métis compared to the general population: ACS 5.3% vs. 3.0%; CHF 5.1% vs. 3.9%; stroke 1.4% vs. 1.1%; atrial fibrillation 2.1% vs. 1.4%; hypertension 34.9% vs. 29.8%. Incident ACS, stroke, and atrial fibrillation were also higher (p<0.05) among the Métis: ACS 2.4% vs. 1.5%; stroke 0.8% vs. 0.6%; atrial fibrillation 0.6% vs. 0.3%. One-year all-cause and cardiovascular-related mortality were not significantly different. Hospitalizations were higher for Métis persons with CHF (OR 1.93; 95% CI 1.34–2.78) and hypertension (OR 2.27; 95% CI 1.88–2.74). Métis with CHF made more emergency department (ED) visits in the year after diagnosis compared to non-Métis with CHF, while Métis aged ≥65 with ACS were more likely to be on beta-blockers following diagnosis.

**Conclusions:**

The burden of cardiovascular disease was markedly higher in the Métis compared to the general population: prevalence rates for five cardiovascular conditions were 25% to 77% higher. Métis persons with CHF had more frequent hospitalizations and ED visits following their diagnosis.

## Introduction

One-third of all Aboriginal people in Canada are Métis, with approximately 452,000 persons self-identifying in the 2011 Canadian National Household Survey [1]. The Métis population is also one of the fastest growing populations in Canada, having doubled between 1996 and 2006 [2]. The Métis trace their ancestry to the offspring of European men and First Nations women, and are a distinct Aboriginal people, set apart from First Nations and Inuit by language, culture, way of life, and history. Although many Métis live in rural and remote areas [3], Métis do not have government-designated tracts of land (reserves), as First Nations do. Métis fall under a different legislative and regulatory structure than First Nations and Inuit, and consequently receive significantly fewer local, provincial, and national resources for health care than do other Aboriginal groups [4]. For example, Métis do not have access to comprehensive prescription drug coverage and other supports housed within the federally funded Non-Insured Health Benefits program.

Despite a growing population and potential inequity in health care compared to other Aboriginal populations in Canada, relatively little research has examined the health status of the Métis, though several gaps in Métis-related health research have recently been identified, particularly for chronic diseases [4–6]. Cardiovascular disease, including heart disease and stroke, is the second leading cause of mortality in Canada, accounting for 20% of all deaths in 2011 [7]. Cardiovascular disease is largely preventable, and treatment can relieve symptoms, improve quality of life, and markedly decrease the risk of death [8]. There is substantial evidence to suggest that cardiovascular disease rates, risk factors, and related mortality are significantly higher in Aboriginal Canadians compared to other Canadians [9–12]; however, there has been little cardiovascular disease research specifically in the Métis population. One study reported a significantly higher prevalence of ischemic heart disease (10.3% versus 7.3%) and hypertension (27.9% versus 24.8%) in Manitoba Métis compared to other Manitobans [13]. Statistics Canada has reported that cardiovascular diseases comprise a large proportion of Potential Years of Life Lost (PYLL) among both Métis men and women nationally [14]. No studies have examined cardiovascular-related outcomes in detail, such as hospitalizations and quality-of-care outcomes, in Métis with cardiovascular disease.

Ontario is Canada’s most populous province and has the highest number of Aboriginal residents [3]. In this study we sought to examine the burden of five cardiovascular diseases in Ontario Métis, in comparison to rates in the rest of the Ontario population: 1) acute myocardial infarction and unstable angina (also termed acute coronary syndromes or ACS); 2) congestive heart failure (CHF); 3) cerebrovascular disease (stroke); 4) atrial fibrillation; and 5) hypertension. We hypothesized that cardiovascular disease prevalence, incidence, and adverse outcomes would be significantly higher in the Métis cohort compared to the rest of the Ontario population.

## Materials and Methods

### Study Design

This retrospective cohort study was commissioned by the Métis Nation of Ontario (MNO) and conducted at the Institute for Clinical Evaluative Sciences in Ontario, Canada. Ethics approval was given by Sunnybrook Health Sciences Centre, and work was conducted according to the Tri-Council Policy guidelines for research involving Aboriginal peoples of Canada [15], as well as MNO’s internal guidelines for collaborative research with external partners. This study used routinely collected, encoded health information from the province of Ontario (copies of which are held at the Institute for Clinical Evaluative Sciences) and did not require informed consent from participants. The Institute for Clinical Evaluative Sciences is named as a prescribed entity under section 45 of the Personal Health Information Protection Act (Ontario Regulation 329/04, Section 18). Under this designation, it can receive and use personal health information in an encoded or de-identified manner without consent for purposes of analysis and compiling statistical information about the health care system of Ontario.

### Study Population

The Métis population examined in this study was identified using the Citizenship Registry of the MNO. The MNO is the main representative body for Métis persons in Ontario; healthcare of the Métis is a primary interest of the organization. Citizenship in the MNO is restricted to those who can supply genealogical documentation and proof of Métis ancestry [16]. Based on the 2011 National Household Survey, the MNO Citizenship Registry represents approximately 17% of the total Métis population of Ontario [1]. Persons aged less than 20 were excluded from this study, in order to restrict the analysis to adult cardiovascular disease.

### Data Sources

The MNO Citizenship Registry as of July 2006 was provided to the Institute for Clinical Evaluative Sciences and, using encoded Ontario health card numbers, individuals in the registry were linked with the Registered Persons Database, a provincial registry of all persons with an Ontario health card number [17]. Ontario provides full health care coverage for medically necessary care (universal health care coverage), so the database includes the vast majority of Ontarians. Those Métis who were successfully linked to the Registered Persons Database and alive as of April 1, 2006 constituted the Métis population in this study. All other Ontario residents who met the same criteria were considered to be part of the Ontario general population.

Encoded health card numbers were used to link both populations to other Ontario healthcare administrative databases. The Ontario Health Insurance Program (OHIP) is the universal health care plan for Ontario residents and the OHIP database contains all fee-for-service billing claims by physicians for consultations (regardless of the setting) and procedures (e.g. echocardiograms). The Canadian Institute for Health Information (CIHI) National Ambulatory Care Reporting System contains abstracted information on all emergency department visits in Ontario. The CIHI Discharge Abstracts Database records information about every hospitalization in Ontario, including diagnoses and procedures performed. The Ontario Drug Benefits database contains claims for prescription drugs received for Ontario residents aged 65 and over, thus analyses using this database were restricted to those over the age of 65 (e.g. beta-blocker use in patients with ACS). Finally, the Ontario Hypertension Database identifies Ontario patients with hypertension based on diagnostic codes in health care records from the above databases using a validated algorithm [18].

### Diagnostic Definitions

Cardiovascular disease cases for the five diseases of interest were identified using the aforementioned databases. The coding of cardiovascular diseases in CIHI has been shown to be of high quality [19]. Specific coding definitions, along with positive predictive value and sensitivity of the codes (where available), and related citations are shown in Table 1.

**Table 1:** Cardiovascular disease types and corresponding definitions in province-wide health administrative databases.

DISEASE	CASE DEFINITION	ICD-9 CODES (OHIP)	ICD-10 CODES (DAD & NACRS)	PPV	Sensitivity
**Acute coronary syndromes (ACS)** (acute myocardial infarction + unstable angina)	DAD (1 MRD in any study year)	-	I21, I22, I23, I24.9, I20.0, I20.	N/A	N/A
**Congestive heart failure (CHF)**	DAD (1 MRD in any study year) *or* OHIP + 2^nd^ OHIP (2 claims in any study year) *or* NACRS + 2^nd^ NACRS (2 main diagnosis visits in any study year)	428	I50	55.6%^1^	84.8%^1^
**Cerebrovascular disease (stroke)**	DAD (1 MRD in any study year)	-	I60, I61, I63, I64	N/A	N/A
**Atrial fibrillation**	DAD (1 MRD in any study year) *or* OHIP + 2^nd^ OHIP (2 claims in any study year) *or* NACRS + 2^nd^ NACRS (2 main diagnosis visits in any study year)	-	I48	93.0%^2^	96.6%^2^
**Hypertension**	DAD (1 record in any study year) *or* OHIP + 2^nd^ OHIP (2 claims in 2 study years) *or* OHIP + DAD (2 claims/records in 2 study years)	401, 402, 403, 404, 405	I10, I11, I12, I13, I15	87%^3^	72%^3^

ICD: International Classification of Diseases, version 9 or 10; OHIP: Ontario Health Insurance Plan; DAD: Discharge Abstract Database; NACRS: National Ambulatory Care Reporting System; PPV: Positive predictive value; MRD: most responsible diagnosis; N/A: Not available

^1^ A similar validated algorithm published (using [OHIP + DAD claim] in place of [NACRS + 2^nd^ NACRS (2 claims in any study year)]: Schultz SE, Rothwell DM, Chen Z, Tu K. Identifying cases of congestive heart failure from administrative data: a validation study using primary care patient records. Chronic Dis Inj Can 2013; 33(3): 160–6.

^2^ Single NACRS I480 main diagnosis: Atzema CL, Austin PC, Miller E, Chong AC, Yun L, Dorian P. A population-based description of atrial fibrillation in the emergency department, 2002–2010. Ann Emerg Med 2013;62(6):570–7

^3^ Validated algorithm published at: Tu K, Campbell NR, Chen Z, Cauch-Dudek K, McAlister FA. Accuracy of administrative databases in identifying patients with hypertension. Open Med 2007; 1(1): 18–26.

### Data Measures

Demographic measures, including age and sex, were obtained for both the Métis and the Ontario general population from the Registered Persons Database. Geographic measures, including neighbourhood income quintile and geographic location by Ontario Local Health Integration Network region, were generated using the Statistics Canada Postal Code Conversion File [20].

### Outcome Measures

Prevalence and incidence for the five cardiovascular diseases were measured in both the Métis and the Ontario general population over a five-year period (fiscal years 2006/07 to 2010/11). Incident cases were created by excluding cases with an entry in the study databases in the previous five years. Age at disease onset (median and interquartile range) was also calculated for each incident disease group. Secondary outcome measures were examined using the incident cohort for each cardiovascular disease and included one-year disease-specific hospitalizations and one-year all-cause and cardiovascular-related mortality for each disease. We also examined several validated quality-of-care measures for four of the cardiovascular disease conditions, again using the incident cohorts: (1) among patients with an incident diagnosis of hypertension, rate of emergency department visits in the subsequent year for hypertension (primary diagnosis) [21, 22]; (2) among patients with an incident diagnosis of CHF, rate of emergency department visits in the subsequent year for CHF (primary diagnosis) [23]; (3) among patients with an incident diagnosis of atrial fibrillation, proportion of patients who receive an outpatient echocardiogram within 6 months of diagnosis [24]; and (4) among patients with an incident diagnosis of ACS, proportion using beta-blockers within 3 months of diagnosis (patients aged 65 and older) [25].

### Data Analysis

Counts and proportions were generated for all demographic variables. Age- and sex-adjusted prevalence, incidence, and one-year mortality rates were generated for both groups (Métis and the general Ontario population). Adjustment was done using model-based indirect standardization: first the presence (vs. absence) of each cardiovascular disease was regressed on age and sex using a logistic regression model of the entire study cohort. From the estimated model, the predicted probability of a cardiovascular disease diagnosis was obtained for each patient. For a given cardiovascular diagnosis, the predicted probabilities were then summed within each of the Métis and the rest of the population to determine the expected number of diagnoses. Finally the model-based indirectly standardized rate was calculated as the observed number of diagnoses in the group divided by the expected number of diagnoses in that group, and multiplied by the observed number of diagnoses in both groups (i.e. [observed or crude rate / expected rate] * observed rate in the overall combined population) [26]. Statistically significant differences between the age- and sex-standardized groups were determined using logistic regression, where the presence of cardiovascular disease was regressed on age, sex, and group. To compare hospital admission rates for each cardiovascular disease between the Métis and the rest of the population, Poisson regression was utilized to regress admission rates over the five year study period on age, sex, and group, using the incident cohort for each disease. A similar approach was employed to compare hypertension and heart failure quality of care outcomes (emergency visits in the year after incident diagnosis) between the groups. To compare differences in quality of care for atrial fibrillation (echocardiography performance within 6 months of incident diagnosis) and ACS (beta-blocker use within 3 months of incident diagnosis), a simple chi-square test was used to compare the proportion of each group who received the intervention. All analyses were performed with SAS software (Version 9.3, SAS Institute Inc., Cary, NC).

## Results

**Fig 1:**
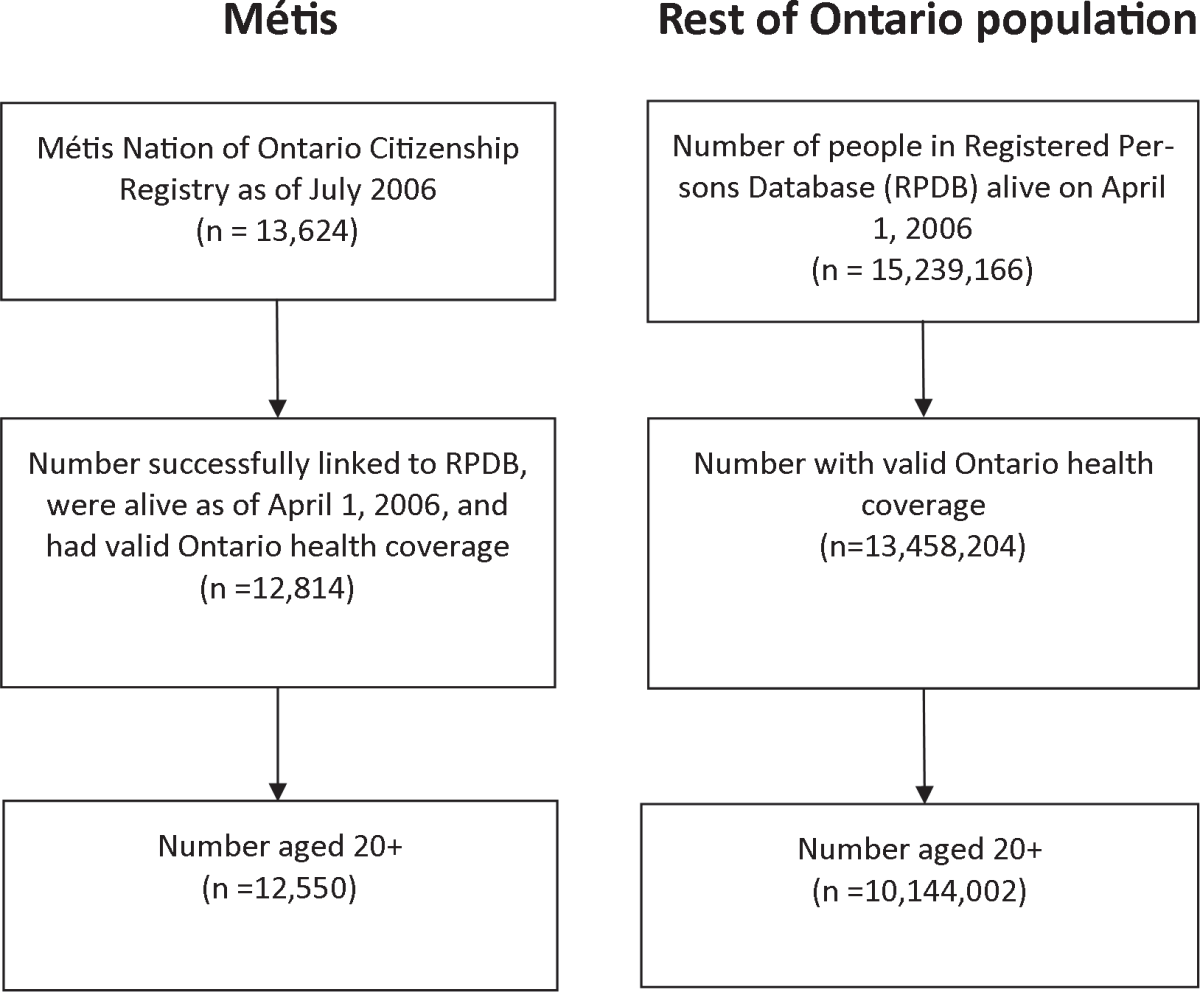
Study flow diagram of Métis and the general Ontario population.

Of the 13,624 individuals in the 2006 MNO Citizenship Registry, 12,814 (94.1%) were successfully linked to the Registered Persons Database, of whom 12,550 (92.1%) were aged 20 and over (Fig. 1). Compared to the rest of the Ontario population, Métis individuals were slightly younger, more likely to be male, more likely to live in lower-income neighbourhoods, and more likely to reside in the North of the province, with the largest group in the North East Local Health Integration Network region (Table 2).

**Table 2:** Demographic characteristics of the Métis and the general Ontario population as of April 1, 2006.

CHARACTERISTIC	MÉTIS (n = 12,550)	ONTARIO (n = 10,144,002)
**Median age (IQR)**	44.0 (32.0–54.0)	45.0 (34.0–58.0)
**Mean age (standard deviation)**	44.2 (14.7)	47.1 (17.1)
**Sex (%)**		
Female	46.4	51.1
Male	53.6	48.9
**Neighbourhood income quintile** ^**1**^ **(%)**		
1 (lowest)	22.7	20.2
2	20.7	20.1
3	20.3	19.5
4	18.0	19.5
5 (highest)	16.7	19.6
missing	1.6	1.1
**Geographic location as per Local Health Integration Network (LHIN) (%)**		
Erie St. Clair	3.3	5.0
South West	3.7	7.1
Waterloo Wellington	2.3	5.3
Hamilton Niagara Haldimand Brant	6.4	10.6
Central West	1.2	5.7
Mississauga Halton	1.8	8.2
Toronto Central	2.4	9.9
Central	2.2	12.6
Central East	4.7	11.6
South East	3.4	3.8
Champlain	5.2	9.5
North Simcoe Muskoka	17.2	3.2
North East	28.5	4.6
North West	16.5	1.9
missing	1.1	0.9

IQR: Interquartile Range

^1^Income quintile was determined from postal codes obtained from the Registered Persons Database and neighbourhood-level median household income from Statistics Canada census data. Quintiles range from poorest (Q1) to wealthiest (Q5).

Between 2006/07 and 2010/11, the age- and sex-adjusted prevalence of cardiovascular disease was significantly higher in the Métis compared to the rest of the Ontario population (Table 3). Hypertension was the most prevalent cardiovascular condition in the Métis, affecting 35% of Métis; the rate was a relative 17% higher than in the rest of the population. Adjusted prevalence of ACS was 77% higher among the Métis cohort compared to the rest of the Ontario population, while for CHF, stroke, and atrial fibrillation, prevalence was 40%, 25%, and 46% higher, respectively, than in the rest of the Ontario population. The age- and sex-standardized incidence of ACS, stroke, and atrial fibrillation over the five study years were also significantly higher in the Métis cohort compared to the general Ontario population: a relative 57%, 36%, and 94% higher during the study period, respectively. The incident rates of CHF and hypertension were not statistically different.

**Table 3:** Age- and sex-adjusted prevalence and incidence of cardiovascular diseases, per 100 persons, in the Métis and the general Ontario population, April 1 2006 to March 31 2011.

DISEASE	MÉTIS(n = 12,550)	ONTARIO(n = 10,144,002)	p-value
**Acute coronary syndromes (ACS)**			
Prevalence (95% CI)	5.27 (4.93–5.60)	2.97 (2.96–2.98)	<0.001*
Incidence (95% CI)	2.37 (2.13–2.61)	1.51 (1.50–1.51)	<0.001*
**Congestive heart failure (CHF)**			
Prevalence (95% CI)	5.14 (4.73–5.54)	3.68 (3.67–3.69)	<0.001*
Incidence (95% CI)	1.35 (1.11–1.59)	1.24 (1.23–1.24)	0.36
**Cerebrovascular disease (stroke)**			
Prevalence (95% CI)	1.37 (1.15–1.59)	1.10 (1.09–1.11)	0.02*
Incidence (95% CI)	0.79 (0.63–0.95)	0.58 (0.57–0.58)	0.01*
**Atrial fibrillation**			
Prevalence (95% CI)	2.08 (1.82–2.34)	1.42 (1.41–1.43)	<0.001*
Incidence (95% CI)	0.62 (0.50–0.73)	0.32 (0.32–0.32)	<0.001*
**Hypertension**			
Prevalence (95% CI)	34.87 (34.04–35.70)	29.76 (29.74–29.79)	<0.001*
Incidence (95% CI)	6.89 (6.46–7.33)	6.56 (6.55–6.58)	0.14

CI: Confidence Interval; IQR: Interquartile Range

* p <0.05

There were no significant differences between study groups in all-cause and cardiovascular-related mortality one year after incident diagnosis (Table 4), although there was a trend toward higher all-cause mortality in Métis with atrial fibrillation.

**Table 4:** Age- and sex-adjusted all-cause and cardiovascular-related^1^ one-year mortality (from incident diagnosis) by cardiovascular disease, per 100 persons, in the Métis and general Ontario population, April 1 2006 to March 31 2012.

DISEASE	MÉTIS (95% CI)	ONTARIO (95% CI)	p-value
**Acute coronary syndromes (ACS)**			
Raw number of incident cases	231	152,959	
Raw number of deaths	18	24,834	
Adjusted all-cause mortality	11.5 (5.7–17.2)	16.2 (16.1–16.4)	0.11
Adjusted cardiovascular mortality	9.8 (4.7–14.8)	11.4 (11.3–11.6)	0.51
**Congestive heart failure (CHF)**			
Raw number of incident cases	111	125,580	
Raw number of deaths	17	18,552	
Adjusted all-cause mortality	25.2 (13.1–37.3)	22.80 (22.5–23.1)	0.70
Adjusted cardiovascular mortality	13.0 (6.5–19.5)	9.3 (9.1–9.5)	0.27
**Cerebrovascular disease (stroke)**			
Raw number of incident cases	66	58,500	
Raw number of deaths	12	13,342	
Adjusted all-cause mortality	20.7 (13.0–28.5)	14.8 (14.6–15.0)	0.13
Adjusted cardiovascular mortality	25.4 (13.4–37.3)	21.8 (21.4–22.1)	0.55
**Atrial fibrillation**			
Raw number of incident cases	56	32,387	
Raw number of deaths	6	2,538	
Adjusted all-cause mortality	16.6 (7.3–25.4)	7.8 (7.5–8.1)	0.06
Adjusted cardiovascular mortality	10.0 (2.4–17.7)	4.8 (4.6–5.0)	0.19
**Hypertension**			
Raw number of incident cases	858	665,868	
Raw number of deaths	11	8,364	
Adjusted all-cause mortality	1.8 (0.89–2.6)	1.3 (1.2–1.3)	0.26
Adjusted cardiovascular mortality	0.82 (0.24–1.4)	0.54 (0.52–0.56)	0.35

CI: Confidence Interval

^1^ ICD 9/10 diagnostic codes to define cardiovascular-related mortality were obtained from: Statistics Canada. Comparability of ICD-10 and ICD-9 for Mortality Statistics in Canada. Ottawa ON, 2005. ICD-9 codes: 390–448. ICD-10 codes: I00-I78.

Differences in disease-specific hospital admission rates one year following an incident diagnosis varied by condition (Table 5). Rates of hospital admissions for hypertension and CHF were twice as high among Métis compared to other Ontarians with these conditions (p<0.001). Hospital admission rates for ACS, stroke, and atrial fibrillation were not statistically different between the Métis and the general population.

**Table 5:** Age- and sex-adjusted rate ratios of one-year disease-specific hospitalizations (from incident diagnosis) by cardiovascular disease, in the Métis and general Ontario population, April 1 2006 to March 31 2012.

DISEASE	RATE RATIO (95% CI)	p-value
Acute coronary syndromes (ACS)	1.23 (0.95–1.61)	0.12
Congestive heart failure (CHF)	1.93 (1.34–2.78)	<0.001*
Cerebrovascular disease (stroke)	1.03 (0.54–1.98)	0.93
Atrial fibrillation	1.23 (0.73–2.08)	0.44
Hypertension	2.27 (1.88–2.74)	<0.001*

CI: Confidence Interval

* p <0.05

For the quality of care measures, Métis persons with ACS had significantly better rates of beta-blocker use after incident diagnosis than other Ontario residents with ACS (Table 6). However, Métis with CHF had twice as many disease-specific emergency department visits in the year following incident diagnosis compared to the general population with CHF (p<0.001). The quality of care outcome for atrial fibrillation (echocardiography) was not significantly different between the two groups, while disease-specific emergency department visits for hypertension displayed a trend (p = 0.08) toward higher rates of visits among Métis.

**Table 6:** Quality of care measures after incident cardiovascular disease diagnosis in the Métis and the rest of the Ontario population, April 1 2006 to March 31 2011.

**POPULATION**	**PERCENTAGE (95% CI)**	**p-value**
**Acute coronary syndromes (ACS)** ^**1**^		
Métis	77.8 (67.8–85.9)	**0.03** *
Ontario	66.9 (66.6–67.3)	
**Atrial fibrillation** ^**2**^		
Métis	52.9 (38.5–67.1)	0.12
Ontario	42.1 (41.5–42.6)	
	**RATE RATIO (95% CI)**	**p-value**
**Congestive heart failure (CHF)** ^**3**^		
Métis vs. Ontario	2.03 (1.47–2.80)	**<0.001** *
**Hypertension** ^**3**^		
Métis vs. Ontario	1.51 (0.95–2.40)	0.08

CI: Confidence Interval

^1^ Defined as frequency of beta-blocker use within 3 months after incident diagnosis in persons aged 65+

^2^ Defined as frequency of outpatient echocardiogram within 6 months of incident diagnosis

^3^ Defined as age- and sex-adjusted rate ratio of emergency department visits in the year after incident diagnosis

* p<0.05

## Discussion

This is the first study to examine rates of multiple cardiovascular diseases, associated outcomes, and quality-of-care measures among a Métis cohort. Relative to the rest of the Ontario population, we found markedly higher prevalence and incidence of cardiovascular disease in the Métis. Acute coronary syndromes (ACS) are the second highest killer of Canadians [7] and were 77% more prevalent among the Métis persons compared to the rest of the population in Canada’s most populous province. The prevalence of CHF was 40% higher among the Métis cohort than in the rest of the population. The prevalence of stroke was 25% higher, with atrial fibrillation (which increases the risk of stroke five-fold [27]) almost 50% higher. Hypertension, a common risk factor for ACS and the most frequent etiology of both heart failure and atrial fibrillation [28], afflicted a third of the Métis cohort. The rates highlight a large burden of cardiovascular disease in age- and sex-matched Métis, and in turn appear to highlight a failure of chronic disease prevention in this Métis group.

Our results are consistent with a previous study that found a higher burden of ischemic heart disease in the Métis (12.2%) compared to non-Métis persons (8.7%) in Manitoba [13]. The rates reported in that study were higher than those found in this study (5.3% versus 3.0%, respectively), likely due to different definitions utilized (the Manitoba report included all forms of coronary artery disease, in addition to ACS); however, the relationship between rates in Métis versus non-Métis persons was similar. National survey data has also found a significantly higher proportion of self-reported “heart problems” and hypertension among Métis persons compared to other Canadians [3], consistent with our results. No previous work has assessed rates of atrial fibrillation or CHF in Métis persons, to our knowledge.

We also found an increased rate of new cases of ACS, stroke, and atrial fibrillation in our Métis cohort, relative to the rest of the Ontario population. An elevated incidence of stroke may be secondary to the increased rates of the two major risk factors for stroke, atrial fibrillation and hypertension [27, 28]. Both are treatable conditions [24, 28]: effective medical management of atrial fibrillation and hypertension could potentially curtail the increasing incidence of stroke in the Métis. The elevated prevalence of diabetes in Ontario Métis that was demonstrated in a previous study [29] may have contributed to a higher incidence of ACS. Undiagnosed diabetes may be a particular problem for Métis who live in remote and rural locations [30], due to barriers in accessing primary care services, again potentially contributing to increased ACS among the Métis.

The other major risk factors for ACS, including high cholesterol, smoking, and physical activity levels, were not available for assessment in this study. Nationally, daily smoking prevalence in the Métis is 30%, twice that of the general Canadian population [31]. However, rates of physical activity appear to be higher in the Métis compared to the general population [3, 32]. Future studies are needed to better delineate the risk factors that contribute to the elevated rate of ACS that was found in this study, in order to facilitate future targeted interventions.

Outcomes in patients with cardiovascular disease varied by disease. The finding of higher hospital admission rates for Métis patients with CHF and hypertension suggests worse control of these conditions in this Métis population, and the need for improved chronic disease management. Poor disease control may be secondary to decreased access to universal health care, as the largest proportion of Métis live in the northeast of the province [3], where primary care visits are lowest [33]. Continuity of care, which includes care coordination between primary and specialist care, has been shown to reduce mortality in patients with CHF [34] and atrial fibrillation [35], as well as emergency department visits [36]. Establishing dedicated Métis health centres and/or health directors, similar to those created for other Aboriginal groups [4], is one potential solution.

Our quality-of-care measure for ACS was better among the Métis cohort (aged 65+) compared to the age-matched general population with ACS, with almost 80% of this cohort filling a prescription for evidence-based medication use (beta-blockers) within three months of an incident ACS diagnosis. This suggests that, despite the higher prevalence and incidence of ACS, the Métis are able to access appropriate care in the initial period after an acute myocardial infarction or unstable angina diagnosis. Given that our diagnosis of ACS was based on a hospitalization for such an event, it suggests that Métis with this disease are being initiated on appropriate therapy during and following their hospitalization. However, among Métis with CHF, emergency department use for their disease was significantly higher, suggesting worse disease control in the outpatient setting. This is consistent with previous work in Manitoba Métis, where the Métis had 13% more ambulatory visits and 26% more hospital admissions overall compared to the general population [13].

## Limitations

While health administrative data has many advantages as an efficient method of determining disease burden, it may contain coding errors. However, many of the ICD codes in this study were validated, and we used validated algorithms where possible to identify cardiovascular diseases. As the MNO Citizenship Registry is voluntary, it may not be representative of the entire Métis population of Ontario. Métis individuals who have not registered may differ in demographic, behavioral or clinical terms from those registered with the MNO. However, we adjusted for age and sex in our analyses, making the two groups comparable on these non-modifiable cardiovascular risk factors. The number of one-year deaths in the Métis cohort during the study period was low, resulting in wide confidence intervals; future studies with a longer follow-up period are needed to assess longer-term mortality outcomes.

## Conclusions

This study suggests that Métis persons in Ontario have a markedly higher burden of cardiovascular disease than the general population. New cases of ACS, stroke, and atrial fibrillation are rising faster in the Métis than in the rest of the population, and Métis with heart failure appear to have poor control of their disease in the outpatient setting. Targeted health interventions in this at-risk group, including better provision and coordination of primary and specialist outpatient care, might decrease the burden of these treatable diseases among Ontario’s Métis population.
